# Low-carbon cements review of freeze-thaw tests: Mediterranean corridor proposal

**DOI:** 10.1007/s11356-026-37699-8

**Published:** 2026-03-31

**Authors:** Abigail Jiménez-Franco, David Torrens-Martin, Imren Basar, Lucía J. Fernández-Carrasco

**Affiliations:** 1 ATEM Research Group, Department of Civil and Environmental Engineering, Barcelona TECH, Universitat Politècnica de Catalunya, 08034 Barcelona, Spain; 2 Geosciences Barcelona (GEO3BCN), CSIC, Lluís Solé I Sabarís S/N, 08028 Barcelona, Spain

**Keywords:** Freeze-thaw cycles, Low-carbon cements, Mediterranean environments, Model proposal

## Abstract

The cement industry is a major contributor to global CO₂ emissions, which has led to the development of low-carbon cements. A critical factor in ensuring the durability of these materials is their resistance to freeze-thaw (F-T) cycles, especially as climate change expands the relevance of these tests beyond traditionally cold regions to areas with increasingly variable climates. This review analyzes current testing standards for low-CO₂ cements exposed to F-T cycles, highlighting gaps in performance research in this field. The study proposes a novel testing framework tailored to the specific temperature and humidity fluctuations found in the Mediterranean corridor, with a focus on north-eastern Spain. A thermal curve model was developed based on regional climate data, offering a more realistic assessment of cement durability in the Mediterranean corridor. The findings emphasize the lack of global data in some countries, as well as the fact that most research has been conducted by countries in cold regions, overlooking the rest of the world where freeze-thaw cycles also occur. This underscores the need for future research to address these gaps and to combine durability and sustainability not only in freeze-thaw testing but also in broader construction and cement research.

**Supplementary Information:**

The online version contains supplementary material available at 10.1007/s11356-026-37699-8.

## Introduction

Climate change is an undeniable reality, and addressing its consequences requires an immediate reduction of greenhouse gases (GHG) emissions from anthropogenic activities. The cement industry plays a substantial role in global CO₂ emissions, contributing over 7% of the total emissions (Worrell et al. 2001). In the building materials sector, concrete stands out as the second most consumed material globally, surpassed only by water. According to the Bill and Melinda Gates Foundation (Gates 2019), over the next 40 years ((Kim 2020)–2060), global urban development will require approximately 2 trillion square feet of new floor space to accommodate the surge in buildings and infrastructure. This growing demand for sustainable construction materials underscores the urgent need to develop low-carbon cement formulations and assess their durability and performance.

Consequently, the construction sector is a major contributor to global carbon emissions, primarily due to the production of ordinary Portland cement (OPC) (Belbachir et al. 2016; WBCSD 2016; Gartner and Sui 2018; Jędrzejewska et al. 2023; GCCA 2024). To mitigate climate change, the development and implementation of low-CO₂ cement-based materials, often referred to as eco-cements, keeping acceptable durability features have become essential. One promising strategy is integrating waste into the cement manufacturing process, a method that has shown potential to significantly reduce CO₂ emissions while repurposing resources that would otherwise contribute to waste accumulation (Environment UN et al. 2018; Frías et al. 2021; Torréns-Martín et al. 2023).

Although the use of waste in cement production has been researched for many years, the urgency to further explore this approach is increasingly apparent. The United Nations Environment Program highlights that incorporating waste materials into cement not only cuts down CO₂ emissions during production but also offers an environmentally sustainable way to manage industrial by-products and waste, thus enhancing the overall sustainability of construction practices (Environment UN et al. 2018). We face the challenge of increasing the substitution of natural raw materials with wastes while ensuring long-term structural performance. With this in mind, freeze-thaw (F-T) resistance has been a critical parameter for assessing the durability of mortar and concrete. Since the earliest studies, such as those by Spangler (1933), this factor has consistently been considered essential. F-T cycles can cause significant damage to concrete structures, leading to cracking and deterioration over time. Therefore, evaluating and improving the F-T resistance of concrete is crucial for ensuring long-term durability and structural integrity.

In cold regions, F-T cycles are a major factor contributing to the deterioration of concrete structures, but we must be aware of the changes that are taking place because of climate change, where the effects of temperature changes are being extended to other warmer regions through the day-night effect. Water inside the concrete expands by up to 9% when it freezes, forming micro-ice bodies. The space for this expansion causes hydraulic and tensile stresses to develop in the pores and increase their size. This larger pore space draws in more water, which, upon refreezing, creates greater tensile stresses, leading to further concrete deterioration (Fagerlund 2002). This process highlights the close relationship between pore space, water, and F-T resistance. Recycled Aggregate Concrete (RAC) is particularly vulnerable to F-T cycles due to the presence of old mortar and micro-cracks on the surface of recycled aggregates, which result in a high-water absorption rate (Xiao et al. 2013). Thus, to meet the standards defined for OPC while innovating in low-CO_2_ cement alternatives, it is crucial to maintain or exceed the quality of mechanical properties and durability standards (Naqi and Jang 2019; Poudyal et al. 2021).

**Fig. 1:**
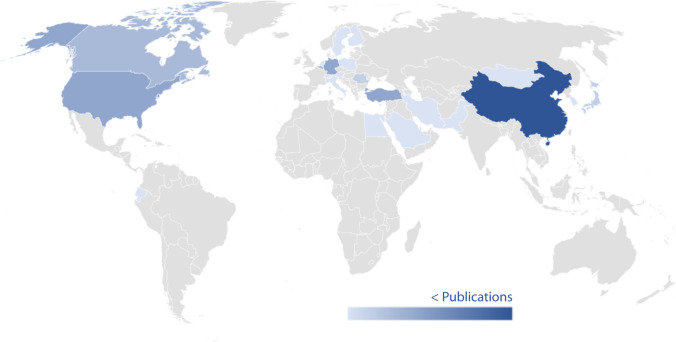
The map shows countries where freeze-thaw resistance is researched for low-CO_2_ cement according to Scopus. Basemap was performed in Microsoft Office® with data of Australian Bureau of Statistics, GeoNames, Geospatial Data Edit, Navinfo, Open Places, OpenStreetMap, TomTom, Wikipedia, and Zenrin

Beyond compressive strength, durability is essential for advancing a more sustainable industry while retaining the benefits of OPC. These properties ensure long-term performance and resistance under various environmental conditions, making the study of low-CO_2_ cement durability under F-T conditions particularly important. In the last two decades, research on low-CO_2_ cement exposed to F-T cycles has increased significantly. As is known, China is one of the main contributors to CO_2_ emissions from cement production and is also one of the leading countries conducting research on this topic. However, also countries in cold regions are focusing on this issue, recognizing F-T testing importance for sustainable construction. Despite the increase in research on this issue, studies testing F-T cycles on low-carbon footprint cement are still scarce (< 40 publications so far this century). Figure 1 shows the countries where F-T resistance has contributed most to the research for low-CO_2_ cement since 2000, according to Scopus. Databases of literature available according to Scopus is shown in Table 1, and Fig. 1 shows that in Europe, territories with colder climates, such as Scandinavia, Germany, and Eastern European nations, are leading the research on F-T resistance in low-CO_2_ cement. This highlights a regional focus where the harshness of winter conditions makes understanding these effects crucial.

**Table 1:** Analyze of publications by country about freeze and thaw tests in 2000–2024 period, listed by Scopus®^1^

Country/territory	Publications
China	8
Germany	4
Türkiye	4
United States	4
Belgium	3
Canada	3
Japan	2
Romania	2
Austria	1
Ecuador	1
Egypt	1
Finland	1
Iran	1
Italy	1
Mongolia	1
Netherlands	1
Pakistan	1
Poland	1
Saudi Arabia	1
South Korea	1
Sweden	1
Switzerland	1
Undefined	1

^1^Results from query of: low-CO_2_, cement, and freeze-thaw keywords from 2000 to 2024

However, this emphasis suggests that the effects of F-T cycles in Mediterranean climates are not being adequately considered. Although Italy has conducted some research on this topic, there remains a gap in addressing how these cycles affect concrete durability in countries with milder but variable climates, such as Spain. This gap calls for more research to better simulate Mediterranean conditions, where temperature variations can still pose durability challenges for cement structures. It should be noted that the Mediterranean region has been recognized as a key climate change hotspot (Giorgi 2006), exhibiting high vulnerability under present conditions and projected to experience higher temperatures along with reduced precipitation throughout the twenty-first century.

**Fig. 2:**
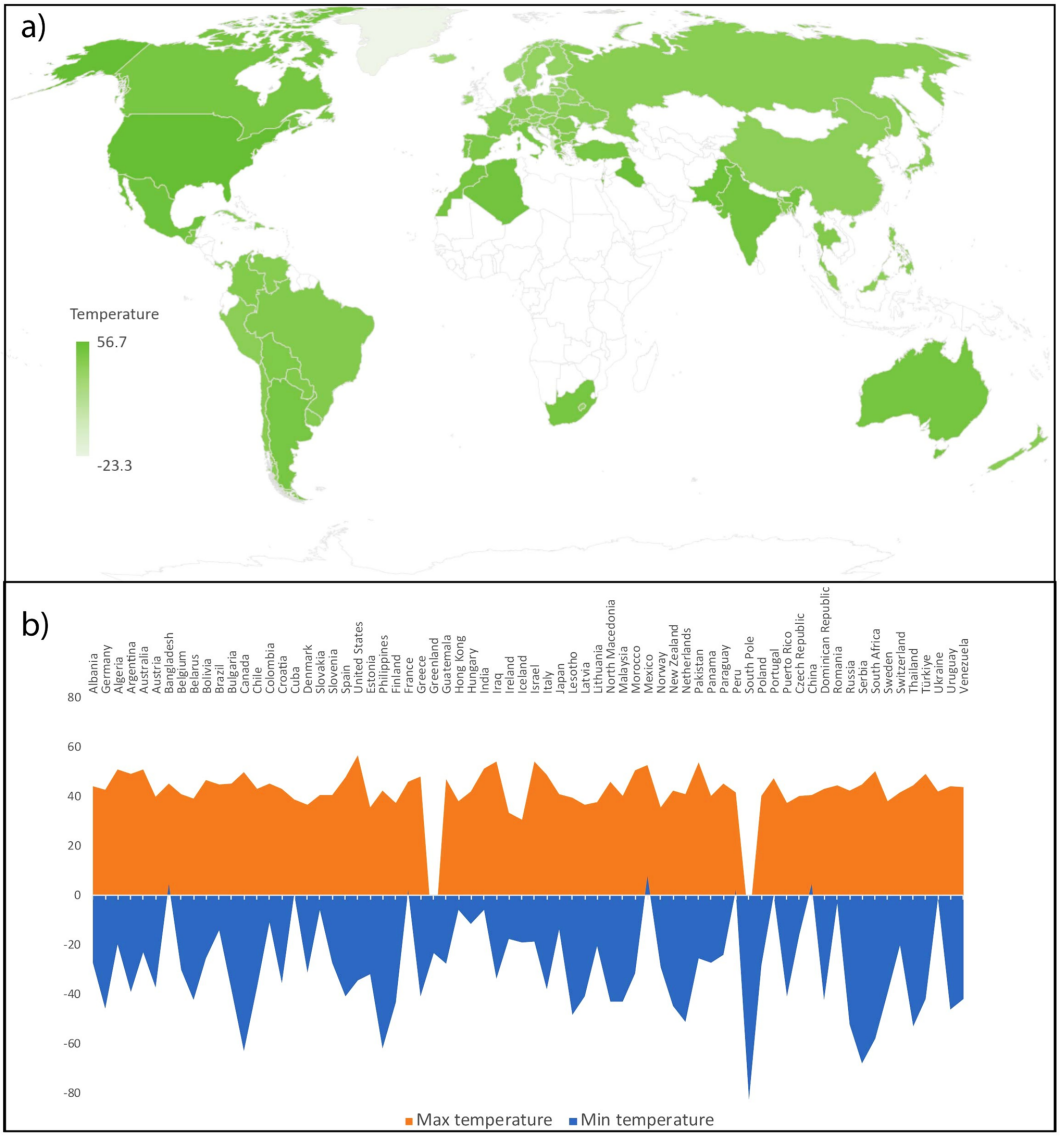
**a** The map shows the countries where freeze-thaw occurs; white color shows the countries without data available. Basemap was performed in Microsoft Office® with data of Australian Bureau of Statistics, GeoNames, Geospatial Data Edit, Navinfo, Open Places, OpenStreetMap, TomTom, Wikipedia, and Zenrin; **b** curve chart of maximum and minimum world’s historical temperatures, with information of Global Climate Report (2022), NCDC.NOAA.gov. National Climatic Data Center (NCDC) of the National Oceanic and Atmospheric Administration (NOAA), and World Meteorological Organization’s World Weather & Climate Extremes Archive)

For the purpose of this paper, a review of the maximum and minimum historical temperature variation of countries currently experiencing freeze-thaw cycles was performed. Figure 2 shows this effect on a global scale. The objective of this research is to find a framework for the experimental determination of freeze-thaw durability tests for cements with low environmental impact in countries where freeze-thaw processes are different from those in cold countries, such as the Mediterranean corridor (north-eastern Spain), and where the current regulations are not so adapted to these situations. This paper reviews the state of the art in freeze-thaw testing carried out in low-carbon cements and proposes a novel test for the Mediterranean case.

## Freeze-thaw cycles: background

As was exposed above, in cold regions, scientific literature shows consensus about F-T cycles being a significant factor in the deterioration of concrete structures due to water expansion during freezing, which creates internal stresses that enlarge the pores and cause further damage as more water infiltrates and refreezes. Although testing this is commonly conducted in cold climates, it should not be limited to these regions, as other areas with fluctuating temperatures could also experience similar damaging effects on concrete, especially in climates where temperature variability is high throughout the year. Initially, testing methods primarily focus on evaluating the frost resistance performance of cement/concrete materials but often fall short of accurately simulating the specific deterioration processes caused by F-T cycles and deicer agents. These methods do not fully replicate the complex interactions and mechanisms involved in concrete deterioration under such conditions, leading to limitations in predicting the actual performance of concrete structures. Consequently, existing protocols may not capture the full extent of damage and reduction in service life experienced by concrete exposed to F-T cycles, highlighting the need for more comprehensive and realistic testing approaches (Xianglin 2009; Xiao et al. 2013; Wang et al. 2022).

F-T cycles deterioration occurs because water inside the concrete expands forming micro-ice bodies. If there is insufficient space for this expansion, hydraulic and tensile stresses develop in the pores, increasing their size. This larger pore space draws in more water, which, upon refreezing, creates greater tensile stresses, leading to further concrete deterioration. This process highlights the close relationship between pore space, water, and freeze-thaw resistance (Guo et al. 2018). Moreover, carbonation can interact with F-T cycles to affect cement resistance, causing significant deterioration in concrete strength and dynamic modulus due to pore structure changes and osmotic pressures (He et al. 2016; Rao et al. 2017). Subsequently, the presence of deicing salts can exacerbate F-T damage in concrete. Mortar specimens exposed to NaCl solutions showed unexpected phase changes and damage even without freezing, indicating complex interactions between salts and cementitious materials (Farnam et al. 2015). In contrast, there is evidence proposing that CO_2_-cured concrete exhibited reduced pore sizes and improved resistance to surface scaling under freeze-thaw cycles in salt solutions (Zhang and Shao 2018). In addition, RAC is particularly vulnerable to F-T cycles due to the presence of old mortar and micro-cracks on the surface of recycled aggregates, which result in a high-water absorption rate (Guo et al. 2018). However, other waste materials used in eco-cements have shown good durability properties (Frías et al. 2018, 2020, 2021; García Giménez et al. 2018; Kabeer and Vyas 2018; Naqi and Jang 2019; Tebbal and Rahmouni 2019; Torres-Carrasco et al. 2019; Li et al. 2020b, 2020a; Brazão Farinha et al. 2021; Sabzi et al. 2021; Thomas and Ślosarczyk 2023; Torréns-Martín et al. 2023; Lee et al. 2023; Mezaouri et al. 2024; Sharifi and Visrudi 2024).

Moreover, recently, several review works have taken a critical overview of how freeze-thaw durability is currently assessed in cementitious systems. These works point out that current F–T testing practices still rely heavily on experimental conditions that do not always correspond to the diverse exposure situations found in service structures. They emphasize that standardized procedures were largely shaped by the requirements of cold regions, typically assuming rapid thermal cycling, high or full degrees of saturation, and repeated severe freezing events. At the same time, they note that the coupled influence of moisture transport processes, humidity fluctuations, and the rate of cooling and heating is often simplified, even though these factors largely govern damage mechanisms in many real environments. A recurring conclusion across these works is that existing standards provide a useful benchmark but are insufficient to represent climates characterized by moderate temperatures and diurnal thermal oscillations (such as Mediterranean regions), where partial saturation and variable humidity are common. This emerging consensus supports the need for climate-adapted testing methodologies such as those explored in the present study (Rajczakowska et al. 2024; Abbas and Muntean 2025; Guler and Akbulut 2025).

## Framework and methodology

Given that F-T cycles can cause significant damage, much of the literature focuses on simulating conditions around the freezing point to reflect those in cold regions. These studies commonly, but not only, employ the ASTM C666 test standard (ASTM International 2008) in its various forms. Even though other authors have found the necessity of testing other ranges of temperature, they made it in lower temperatures (−52.5 °C and −18 °C) (Bumanis et al. 2018).

However, countries with significant temperature variability throughout the year require testing methods that encompass a wider range of temperatures and relative humidity levels. In Spain, the UNE-CEN/TS EX (AENOR 2008) considers a wider range of temperature and humidity conditions, but is inaccurate to the actual change in the climate. Assessing F-T cycles under diverse environmental conditions (a broader interval for humidity and temperature) offers a more accurate evaluation of cement performance not only in cold regions but also in regions with varying climates.

In first place, to gain a current overview of how cement durability is evaluated, a comprehensive literature review was conducted with a specific focus on the standards applied in freeze-thaw (F–T) testing. For this purpose, studies were reviewed according to three criteria: (i) the use of low-CO₂ binders (low-clinker systems or technologically relevant clinker substitutions), (ii) the application of standardized and traceable F–T procedures, and (iii) a clear and traceable description of the freeze-thaw testing procedure. The resulting compilation is presented in Table 2.

**Table 2:** Review of the different F-T standards applied in low-CO_2_ cement

Curate	Country/ region	Temperature (°C)	Humidity (%)	Cycles	Standard	Reference
7	Colombia	25 to –8	—	71 cycles of 24 h	Unknow	Páez Moreno et al. 2007
90	USA	–17.8 ± 2.7; 23 ± 1.7	45 to 55	10 cycles 16–18 h and 6–8 h	SHRP H205.8	Shi et al. 2011
28	Algeria	–18 ± 2; 20	—	24 cycles 48 h and 6 h	NF P 18-425	Tebbal and Rahmouni 2019
-	Iran	–18 to 4	—	200 cycles, 4 cycles per day	ASTM C666	Divanedari and Eskandari-Naddaf 2020
28	Portugal	4 to –18	—	300 cycles, 6 cycles per day	ASTM C666M-03	Ribeiro et al. 2010
91	Japan	20 to –18	—	28 cycles of 24 h	JSCE-C 507- 2018	Ta et al. 2023
28, 31	Spain	20 to –20	65 ± 5	28 cycles of 24 h	UNE-CEN/TS 12390-9 EX	León et al. 2013
28	Taiwan	4.4 to –17.8	—	600 cycles of 3 h	ASTM C666 -97	Lee et al. 2023
35	Spain	20 to –20	15 to 98	29 cycles of 24 h	UNE-CEN/TS 12390-9 EX	Romero Mendoza 2011
7, 28	Cold Regions	20 to –70	—	2 cycles on 24 h	ASTM C666M-03	Kim et al. 2019a
28	China	–18 to 5; –20 to 20	—	2 cycles of 4 h	GB50082-2009	Xianglin 2009; Xiao et al. 2013; Wang et al. 2022
28	Poland	20 to −20	—	up to ~150	CEN/TR 15177	Tałaj et al. 2023; Góra et al. 2024
28, 90	Finland	−18 to +20	—	56–91	CEN/TR 15177	Iqbal 2026
28	United Kingdom / Europe	20 to −20	—	28	United Kingdom / Europe	Adu-Amankwah et al. 2018
90	Cold Regions	4 to –18	—	Several styles	ASTM C666M	Xianglin 2009; Ribeiro et al. 2010; Xiao et al. 2013; Benli et al. 2018; Kim et al. 2019b; Divanedari and Eskandari-Naddaf 2020; Taheri et al. 2021; Wang et al. 2022; Lee et al. 2023
28	Cold Regions	–17 to 23	—	22 cycles of 26 h	ASTM C672-2012	Xianglin 2009; Xiao et al. 2013; Wang et al. 2022
28	Cold Regions	–20 to 20	—	12 h per cycle	RILEM TC176-IDC2002	Xianglin 2009; Xiao et al. 2013; Wang et al. 2022

Table 2 shows that the ASTM C666 standard (ASTM International 2008) and its variations are the most widely used for freeze-thaw testing. 

**Fig. 3:**
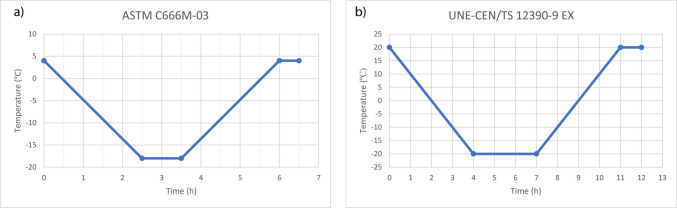
**a** Thermal curve of ASTM C666 standard (ASTM International 2008) and **b** thermal curve of UNE-CEN/TS EX standard (AENOR 2008)

However, this standard has the previously mentioned limitation of a narrow temperature range. In addition to its limited temperature interval, ASTM C666 presents other methodological constraints when applied to Mediterranean climates. First, the standard assumes very rapid cooling and heating rates, which are representative of severe continental and cold-region environments but rarely occur under Mediterranean day–night fluctuations (Supplementary Material). Second, the procedure is based on fully saturated specimens, whereas real structures in temperate climates often experience partial saturation, intermittent wetting and drying, and variable capillary suction. These differences are critical since the rate of cooling, the degree of saturation, and the presence of moisture gradients strongly influence pore pressures, cracking mechanisms, and the progression of freeze-thaw damage. Finally, the standard does not account for humidity fluctuations, which are particularly relevant in regions where F–T cycling frequently coincides with moderate temperatures and changing ambient relative humidity. As several authors have pointed out, these limitations hinder a realistic assessment of durability in environments that do not mimic classical cold-region exposure conditions (Xianglin 2009; Xiao et al. 2013; Wang et al. 2022). After reviewing the literature summarized in Table 2, we also selected the UNE-CEN/TS EX standard (AENOR 2008), which offers an alternative method for measuring moisture conditions using a chamber with controlled temperature, humidity, and time settings. Figure 3 compares the idealized thermal curves of both standards.

**Fig. 4:**
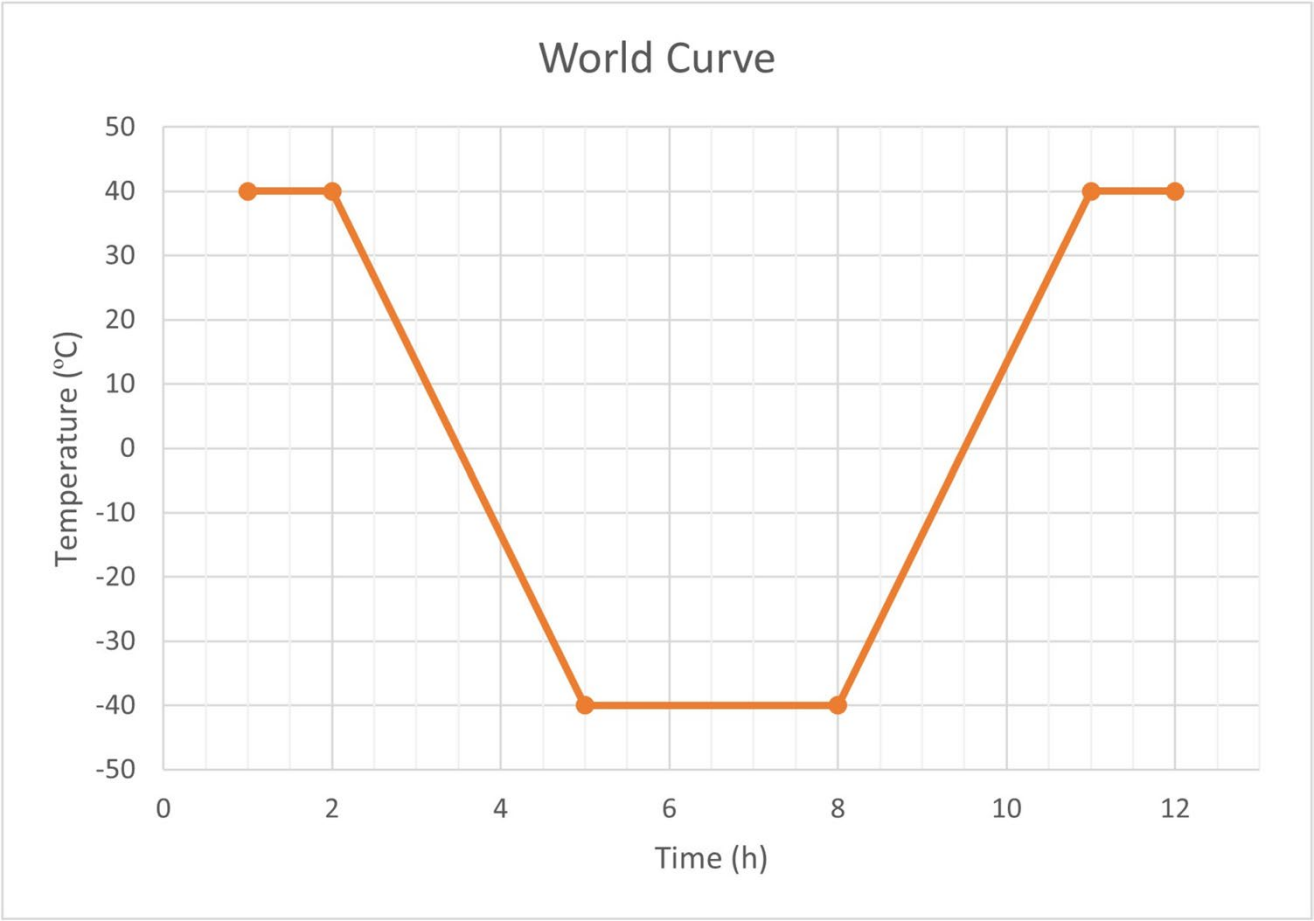
Theoretical proposed freeze-thaw curve for global extremes historical temperatures based on NCDC.NOAA.gov. National Climatic Data Center (NCDC) of the National Oceanic and Atmospheric Administration (NOAA) and World Meteorological Organization’s World Weather & Climate Extremes Archive. This range is designed to account for global extremes and includes regions where historical temperature data are unavailable. Despite the lack of detailed information in certain countries’ records, this interval likely encompasses them, as it reflects the most extreme temperatures observed worldwide

Based on the historical temperature records shown in Fig. 2, freeze-thaw cycles should be evaluated across a broad temperature range of 40 to −40 °C, which seems to better encompass temperature extremes that occur in a wide range of countries and which have not been addressed or covered by existing regulations. Figure 4 presents an analysis curve that is proposed for the countries subject to these temperature ranges, which seems to be more appropriate. Additionally, this proposed range considers the global trend of rising temperatures due to climate change, ensuring that the model remains relevant under future climate scenarios. In the case in point, taking into account the temperature range in the theoretical thermal world curve, we explored and incorporated climate data to determine updated temperature and humidity variations specific to the Mediterranean region, using Catalonia as a representative case study. This localized approach ensures that the study is adapted to regional environmental conditions, complementing the testing standards used and offering a practical application for the Mediterranean climate. By aligning the model with these real-world conditions, we ensure that the proposed methodology addresses the specific needs of Mediterranean climates, providing a more accurate framework for testing freeze-thaw resistance in low-CO_2_ cements.

Considering the aforementioned points, it becomes clear that the current standards predominantly cater to cold climate regions, with the ASTM C666 standard being the most widely used despite its limited temperature range. The UNE-CEN/TS EX standard, offering more comprehensive testing by incorporating temperature and humidity control, stands out as a more flexible option, especially for regions like the Mediterranean, which experience a wider range of environmental conditions. By integrating local Mediterranean thermometric from Catalonia, this study aims to provide a more tailored approach to evaluating cement durability under freeze-thaw conditions. This methodology not only addresses gaps in the existing literature but also underscores the importance of adapting testing standards to diverse environmental contexts.

### Mediterranean climate parameters

*Mediterranean climate* is a category within qualitative global climate classifications (e.g., Köppen 1936) and is used to characterize not only the Mediterranean region but also other, typically smaller areas, including central Chile, southwestern Africa, southwestern Australia, and regions of North America. This kind of climate is generally located on the western coasts of continents between about 30° and 40° latitude. Notwithstanding, the presence of a relatively large mass of water is unique to the actual Mediterranean region.

According to the traditional Köppen classification, the Mediterranean climate is characterized by mild, wet winters and warm to hot, dry summers. Subregional differences can be observed within the Mediterranean Basin due to the differences in the distance to the sea, orography, and latitudes. This work focuses primarily on two climate subtypes—the humid subtropical climate without a dry season and the maritime temperate climate—which can be found in numerous regions around the Mediterranean Sea. Both climate subtypes also occurred in coastal and pre-coastal areas of Catalonia (north-eastern Iberian Peninsula), respectively (Table S1).

The Catalan region, owing to its location at the boundary between the temperate climatic zone to the north and the tropical zone to the south, exhibits characteristics of both domains. During summer, subtropical air masses originating from North Africa prevail, whereas in winter, polar or continental air masses occur more frequently. The presence of the Mediterranean Sea to the east of Catalonia exerts a strong influence on the climate of coastal and pre-coastal regions, moderating thermal extremes and providing a continuous source of atmospheric moisture which, under favorable conditions, contributes to the development of intense rainfall events. In contrast, increasing distance from the sea enhances the influence of continental climatic elements, resulting in more pronounced diurnal temperature contrasts.

Thus, despite its relatively small geographical extent, Catalonia exhibits substantial climatic diversity, such that it cannot be described by a single climatic regime. Coastal and pre-coastal areas of Catalonia are traditionally characterized by mean annual temperatures ranging from approximately 11 to 27 °C, mean annual precipitation totals between 500 and 1000 mm, and mean annual thermal amplitudes of about 14–15 °C in the coastal region and 15–18 °C in the pre-coastal region (Table S1). Precipitation is highly irregular due to the influence of Mediterranean air masses that moderates temperatures while also favoring the occurrence of torrential rainfall events, especially during autumn. Moving inland, thermal and fluviometric characteristics progressively change, with an overall increase in thermal amplitude. Considering that a minimum period of 30 years is generally required to characterize climate conditions, and taking advantage of the data availability for the proposed study area provided by the Catalan weather survey (*Servei Meteorològic de Catalunya, Meteocat),* supported by the Catalan Government, thermometric data for the period 1991–2020 were analyzed. In addition, in order to capture the full range of temperatures observed in the study area, extreme temperature records from *Meteocat* for the period 2020–2024 were also included, possibly reflecting the unprecedented thermal extremes associated with recent global warming.

Since 1991, one of the highest annual absolute maxima temperatures recorded in Catalonia was detected in the south (43.6 °C; Vinebre, Tarragona). Regarding the annual absolute minima, temperature highlights those occurred in Barcelona (−1.1 °C; Fig. S1 & Supplemental data). Considering the most singular meteorological episodes such as heatwaves and cold spells during the most recent years (2020–2024), the summer 2023 heatwave in the northern Catalan coastal area stands out, during which a record temperature of 45.1 °C was recorded, as well as a minimum temperature of − 5.2 °C observed in the southern Catalan coastal area during the same year. In summary, the total range of temperatures for the coastal and pre-coastal areas of Catalonia for the period 1991–2024 according to the explored data is encompassed between 45.1 and −5.2 °C if the most extreme events are included. Regarding the range of the annual mean daily temperature amplitude, for the period 1991–2020, the lowest amplitude corresponds to the central part of the coastal region (5.8 °C) and the highest one to the northern part of the pre-coastal region (14.9 °C; Fig. S1 & Supplemental Data).

**Fig. 5:**
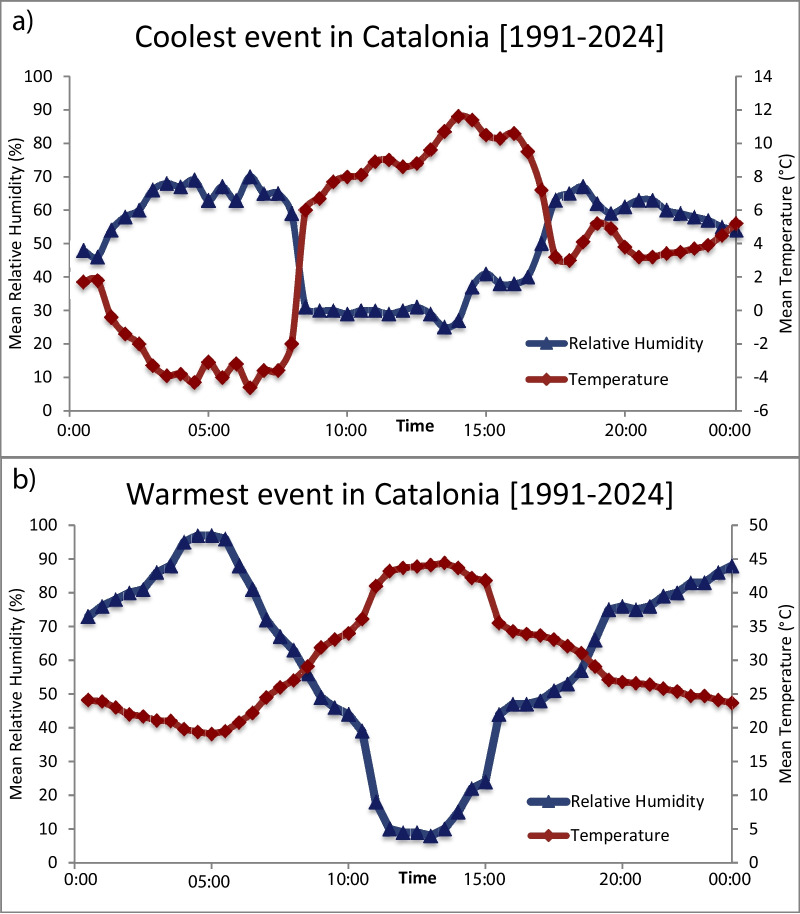
Mean diurnal cycle of air temperature (TM, °C) and relative humidity (HRM, %) during the coldest (**a**) and the warmest (**b**) episodes in Catalonia (1991–2024), showing a midday temperature maximum and a concurrent minimum in relative humidity. The full dataset, including statistical descriptors (mean values, variability, and distributional characteristics), is provided in the Supplementary Data.

The two most singular thermometric events (the 22/01/2023 cold spell and the 18/07/2023 heat wave) took place in the coastal Catalan region (Baix Penedès and Alt Empordà counties, respectively). Through the comparison of the respective daily temperatures and relative humidity, a part of the opposite trends between both parameters, certain cyclicity can be observed, which approximately answers to a 9 h-cycle (Fig. 5).

The Table S2 summarizes hourly warming/cooling rates (°C h⁻^1^) and relative humidity (HRM, %) during the coldest and warmest days recorded in Catalonia in 2023. On the coldest day (22 January 2023, Vendrell, southern Catalonia), the mean thermal change was slightly positive (0.2 °C h⁻^1^), with maximum warming occurring in the early morning and maximum cooling in the late afternoon. Relative humidity showed moderate mean values (57.5%), peaking in the early morning (70%) and reaching minimum levels around midday (25%). In contrast, on the warmest day (18 July 2023, Navata and the Darnius–Boadella Reservoir, northern Catalonia), the mean thermal change was negative (−0.6 °C h⁻^1^). The strongest warming was observed in the morning, while the most intense cooling occurred during the early afternoon. Relative humidity was higher on average (69.5%), with very high nocturnal maxima (97%) and extremely low minimum values around midday (8%), reflecting strong diurnal variability, also in concordance with León et al. (2013) criteria for Spain.

## Results and discussion

### Alternative freeze-thaw testing for Catalonia

In general, scientific literature shows that progress has been made in the development of low-CO_2_ cements. However, while other sectors of industry (e.g., information technology, biotechnology, space exploration, energy, transportation, etc.) have experienced radical innovations, bulk material processing industries, like cement and concrete, have mostly seen incremental advancements. In some cases, disruptive innovations in various industries have been driven by entrepreneurs outside the traditional players. This poses a challenge for the cement and concrete industry to avoid being an exception to this trend. To foster transformative change, a roadmap for radical innovation in the cement value chain must address both challenges and opportunities (van Deventer et al. 2021).

Companies typically prioritize research aligned with immediate production needs, with limited resources allocated to speculative, blue-sky research. Specifically, about the durability of low-CO_2_ cement, several aspects are not resolved yet, such as the phase stability due to the exposure conditions, the relationship between permeability degradation and carbonatation, prevention of alkali-silica reaction, innovative methods of testing durability, and service life prediction (van Deventer et al. 2021). The review of literature made in this work points out the gaps that exist in the realm of testing of F-T cycles beyond the current standards.

Consequently, research on cementitious materials must promote the imperative to shift the cement industry towards low-CO_2_ production. Urgently needed are models predicting concrete/cement service life linked to microstructural phenomena and exposure to real conditions. It must be taken into consideration that different testing methodologies can affect the perceived durability of concrete/cement. Comparisons between different standard tests have resulted in more representative field conditions due, for instance, to continuous water availability and top-down freezing (George and Davidson 1963). Moreover, initially, for the experimental design of F-T cycles it was considered that damage increased with the number of cycles. However, the results of damage progression are also dependent on moisture content. This implied that frost and high moisture levels impact compressive strength, tensile strength, bond strength, and E-modulus of concrete (Fagerlund 2002), not always in a negative way. Fagerlund’s work (2002) pointed out that the determination of the number of freeze-thaw cycles would be on the basis of daily maximum and minimum air temperatures obtained from weather records. In addition, the F-T exposure significantly affects the performance and failure modes of composite-confined concrete, with more catastrophic failures observed in specimens subjected to these cycles (Karbhari et al. 2000).

**Fig. 6:**
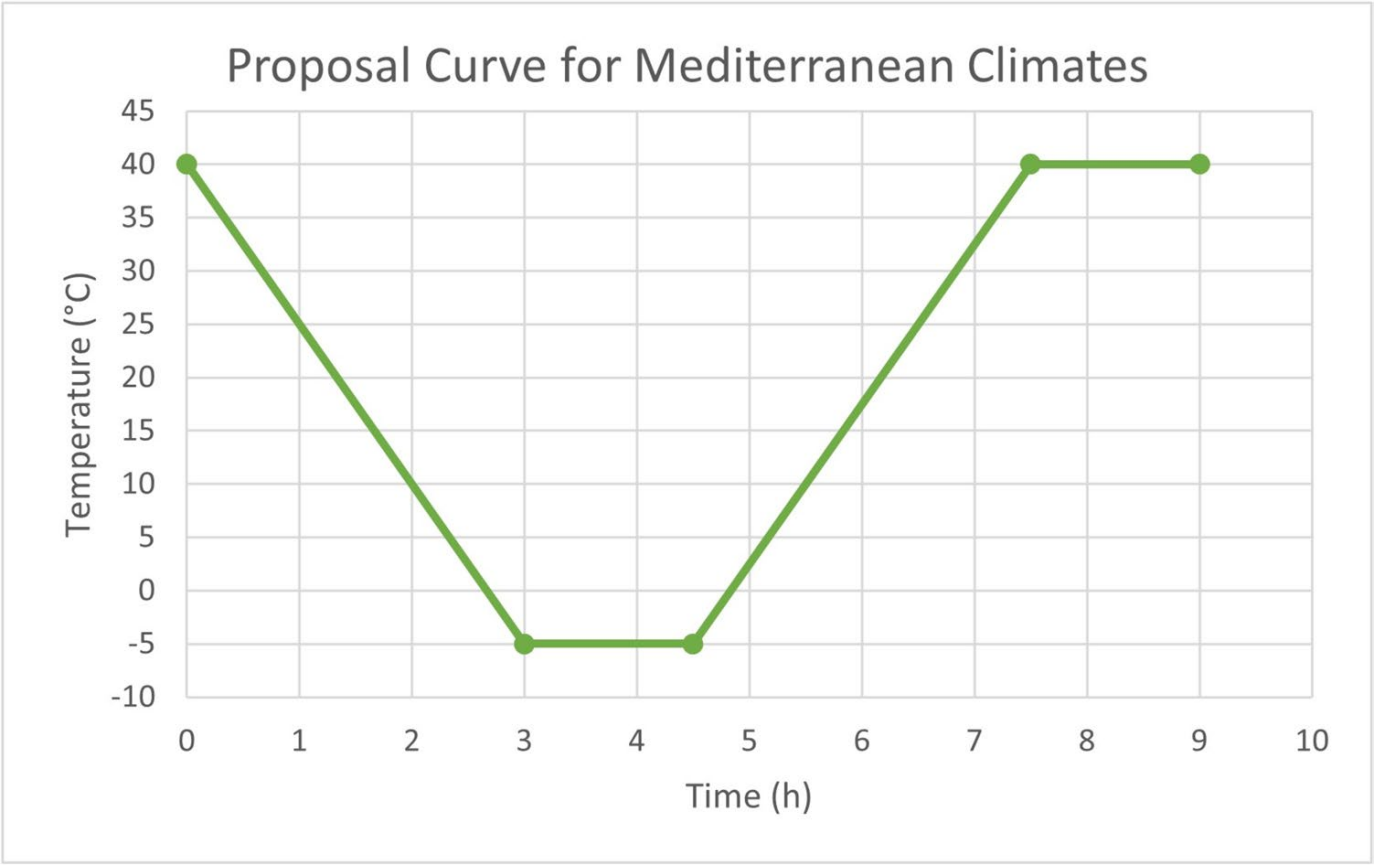
**a** Thermal curve proposed in this study, modified from UNE-CEN/TS EX standard (AENOR 2008), and the relative humidity for this temperature range selected is 70%

In view of the previous discussion and in line with the methodology and data presented in this study (Table 2, Fig. 5, Table S1, Supplemental material & Supplemental data), we derived the theoretical thermal curve shown in Fig. 6 for current Mediterranean climate conditions, using the Catalonia region as a study case. The curve reflects extreme temperatures ranging from −5 to 40 °C, with a relative humidity of 70%. The duration was set to 9 h, following the recommendation of Fagerlund (2002), simulating a more realistic thermal cycle for daily fluctuations in this specific environmental context. Finally, the temperature ramps were performed taking into account the ASTM C666 standard (ASTM International 2008). This approach seeks to address the lack of realistic standards for Mediterranean environments in F-T testing and, more broadly, for the durability of low-CO_2_ cement. The proposed thermal curve can be adapted for different testing cycles, with an initial recommendation of testing between 10 and 50 cycles (10 cycles: early-stage response; 30 cycles: intermediate damage; 50 cycles: advanced deterioration). However, it is essential to consider that an excessively high number of cycles does not necessarily yield more accurate predictions of cement durability. As Fagerlund (2002) pointed out, the availability of water in the exposure environment is an equally important factor in these tests.

This proposal not only aims to establish more accurate testing protocols for Mediterranean climates but also highlights the importance of addressing the underexplored role of humidity in selecting this type of durability assessment.

### Future work and validation plan

The thermal curve proposed in this study constitutes a conceptual framework developed to better represent Mediterranean-type environments; at this stage, however, it remains theoretical and must be experimentally validated. Future work will therefore focus on a structured validation program designed to compare the performance of low-CO₂ cementitious materials subjected to: (i) the proposed Mediterranean curve and (ii) conventional standards.

The experimental program will be organized in three main phases:

Phase 1: Material selection and initial characterization. Low-CO₂ cements will be selected and characterized in the as-received state. This phase will include chemical and mineralogical characterization by X-ray diffraction (XRD) and Fourier-transform infrared spectroscopy (FTIR), as well as optical microscopy to assess clinker phases and early hydration products. These data will establish a baseline to interpret subsequent microstructural changes induced by F–T exposure and other durability tests.

Phase 2: Specimen preparation. Mortars will be prepared according to standard mix designs representative of structural applications. Three curing ages will be considered before F–T testing: 7 days (early-age behavior), 28 days (conventional reference age), and 1 year (long-term microstructural development). For each curing age, specimens will be divided into the exposure groups in order to compare the standards with the proposed Mediterranean curve.

**Fig. 7:**
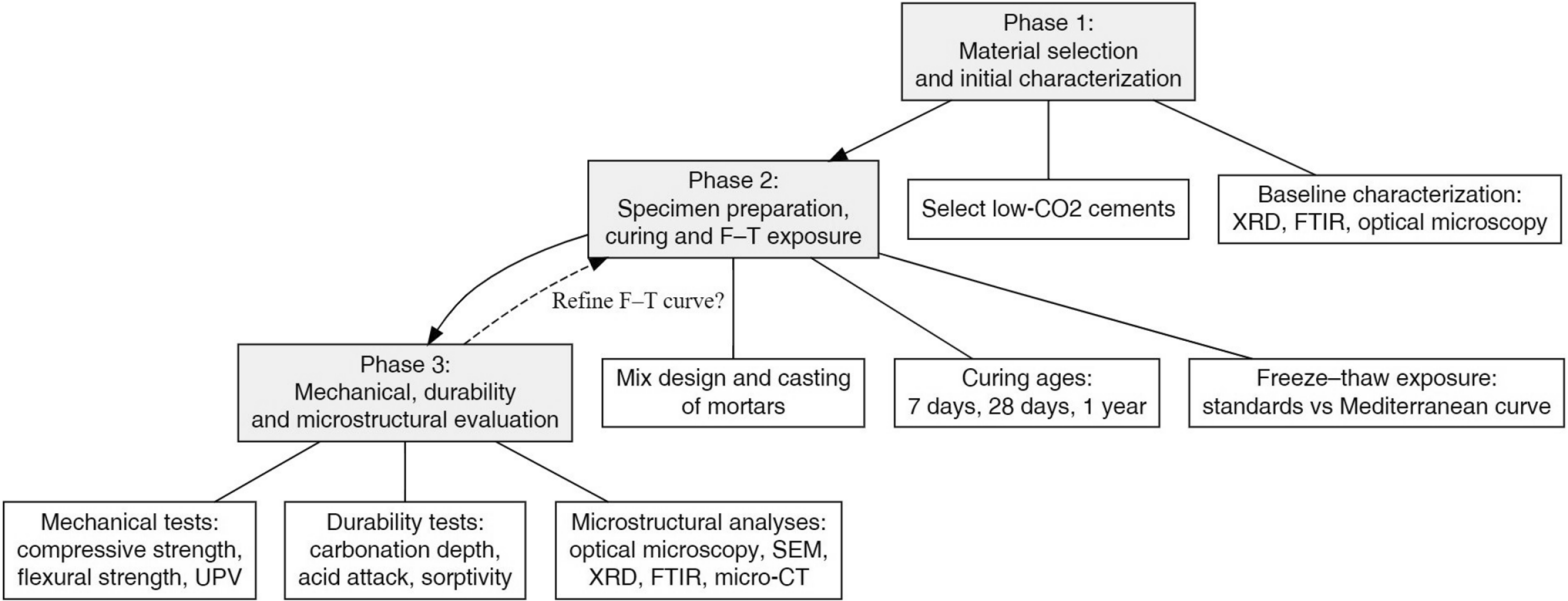
Workflow of the proposed validation plan for the Mediterranean freeze-thaw curve. The scheme summarizes the sequential phases of the study: (i) initial selection and characterization of low-CO₂ binders, (ii) specimen preparation and exposure under conventional and Mediterranean freeze-thaw regimes, and (iii) integrated durability and microstructural evaluation. Feedback loops allow iterative refinement of the proposed thermal curve based on the experimental results

Phase 3: Mechanical, durability and microstructural evaluation. After each F–T block, specimens will be tested using a combined set of performance indicators: mechanical tests (compressive strength, flexural strength and ultrasonic pulse velocity (UPV) to quantify stiffness degradation and internal damage), durability tests (e.g., accelerated carbonation depth, acid attack, etc.). These tests will allow evaluation of whether the proposed F–T curve leads to deterioration patterns consistent with other durability indicators, and microstructural analyses (optical and electron microscopy (SEM-EDS), XRD and FTIR to monitor phase evolution and microcracking, and X-ray micro-computed tomography to quantify internal damage in three dimensions). By comparing these metrics across the three F–T procedures at different curing ages, it will be possible to determine whether the proposed Mediterranean curve reproduces damage mechanisms and durability trends that are more consistent with expected field exposure in Mediterranean climates than those obtained under conventional cold-region standards. The workflow of this proposal is shown in Fig. 7. In this way, the theoretical model presented here can evolve into a robust, experimentally supported protocol for assessing the freeze-thaw durability of low-CO₂ cements in Mediterranean-type environments.

## Conclusions

The shift towards low-CO_2_ cement-based materials is a crucial step in reducing the environmental impact of the construction industry. However, ensuring that these materials can withstand the challenges without compromising durability properties is essential for their widespread adoption. This review highlights the importance of comprehensive testing for freeze-thaw durability, as these cycles can significantly impact the lifespan and structural integrity of cement/concrete, especially in climates with variable temperatures.

Until now, available testing methods are often limited to simulating conditions near the freezing point and may not fully capture the real-world deterioration processes in regions with more varied climate patterns. Expanding the scope of durability testing to include these factors will help improve the reliability of eco-cements, making them more suitable for use in diverse environmental conditions. Addressing these research gaps is crucial to advancing the adoption of low-CO_2_ cements while maintaining performance standards comparable to conventional Portland cement.

Thus, this study presents a novel theoretical thermal curve model as a result of the need identified in the literature review. We propose a standard specifically tailored to Mediterranean climates, such as that of Catalonia, with temperature ranges from −5 to 40 °C, a relative humidity of 70%, and a cycle duration of 9 h. This model addresses the unique environmental conditions of these regions and aims to provide a more accurate framework for testing the durability of conventional and low-CO_2_ cement materials in such climates.

It is important to highlight the necessity of obtaining specific records of extreme temperatures for countries where historical data is limited or unavailable. Additionally, it is essential to insist on the use of models that are updated to reflect current climatic conditions in each region, rather than solely relying on existing standards. By doing so, testing methodologies can better reflect the reality of local climates, ensuring that the durability of conventional and low-CO_2_ cement materials is accurately assessed and adapted to diverse environmental conditions across the globe.

## Supplementary Information

Below is the link to the electronic supplementary material.

ESM 1 (DOCX 1.06 MB)

## Data Availability

The authors declare that the data supporting the findings of this study are available within the paper.
