# Supplementary material for: Low-carbon cements review of freeze-thaw tests: Mediterranean corridor proposal
Source: Environ Sci Pollut Res Int. 2026 Mar 31;33(14):6419–32. doi: 10.1007/s11356-026-37699-8 (PMC13124839; doi:10.1007/s11356-026-37699-8)

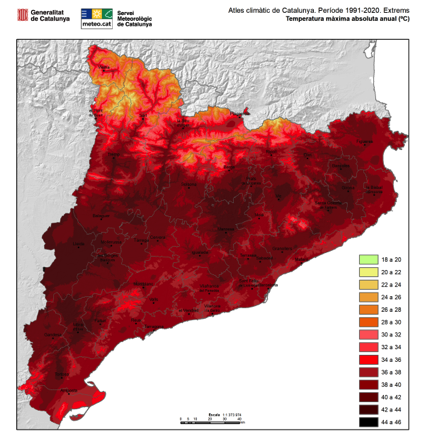

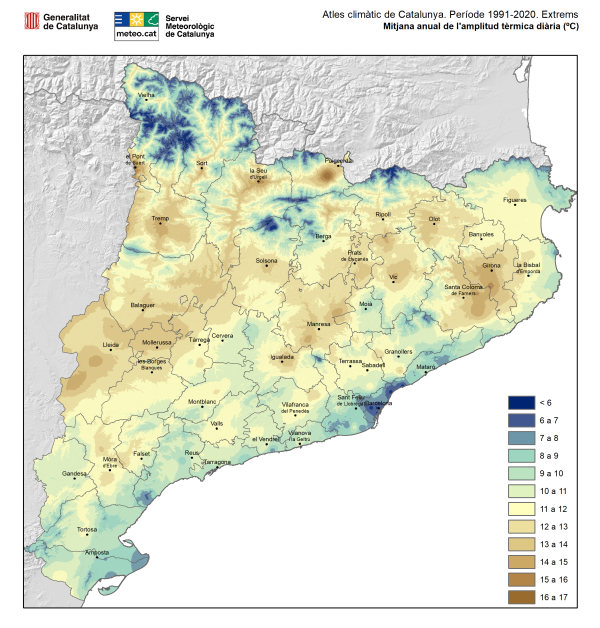

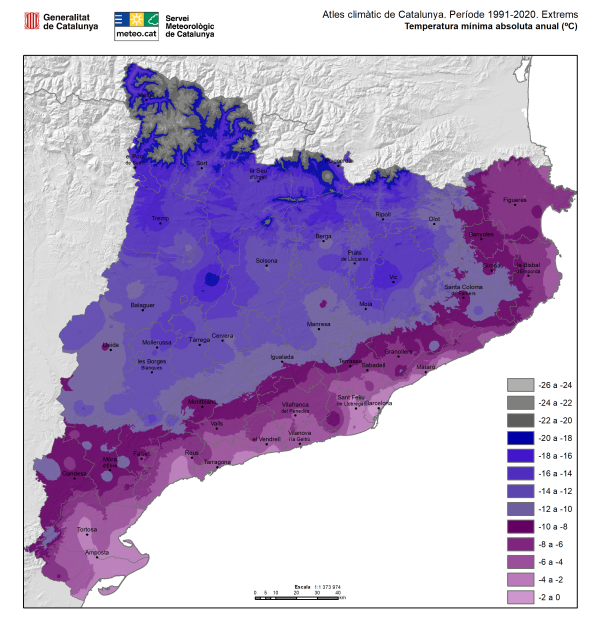


Figure S1. Maps showing climate parameters for Catalonia for the period [1991-2020]. From left to right: Annual absolute máxima temperatura; Annual absolute minima temperatura; Daily thermal amplitude (Meteocat, <https://www.meteo.cat/wpweb/climatologia/el-clima/atles-climatic/atles-climatic-extrems/#municipal>)

# Table S2. Hourly Warming and Cooling Rates and Relative Humidity Variability on Extreme Temperature Days in the coastal and pre-coastal Catalan region; 1991-2024. (Data extracted from Meteocat). The coolest event correspond to 22/01/2023 (-5.2ºC) and the warmest to 18/07/2023 (45.1ºC).


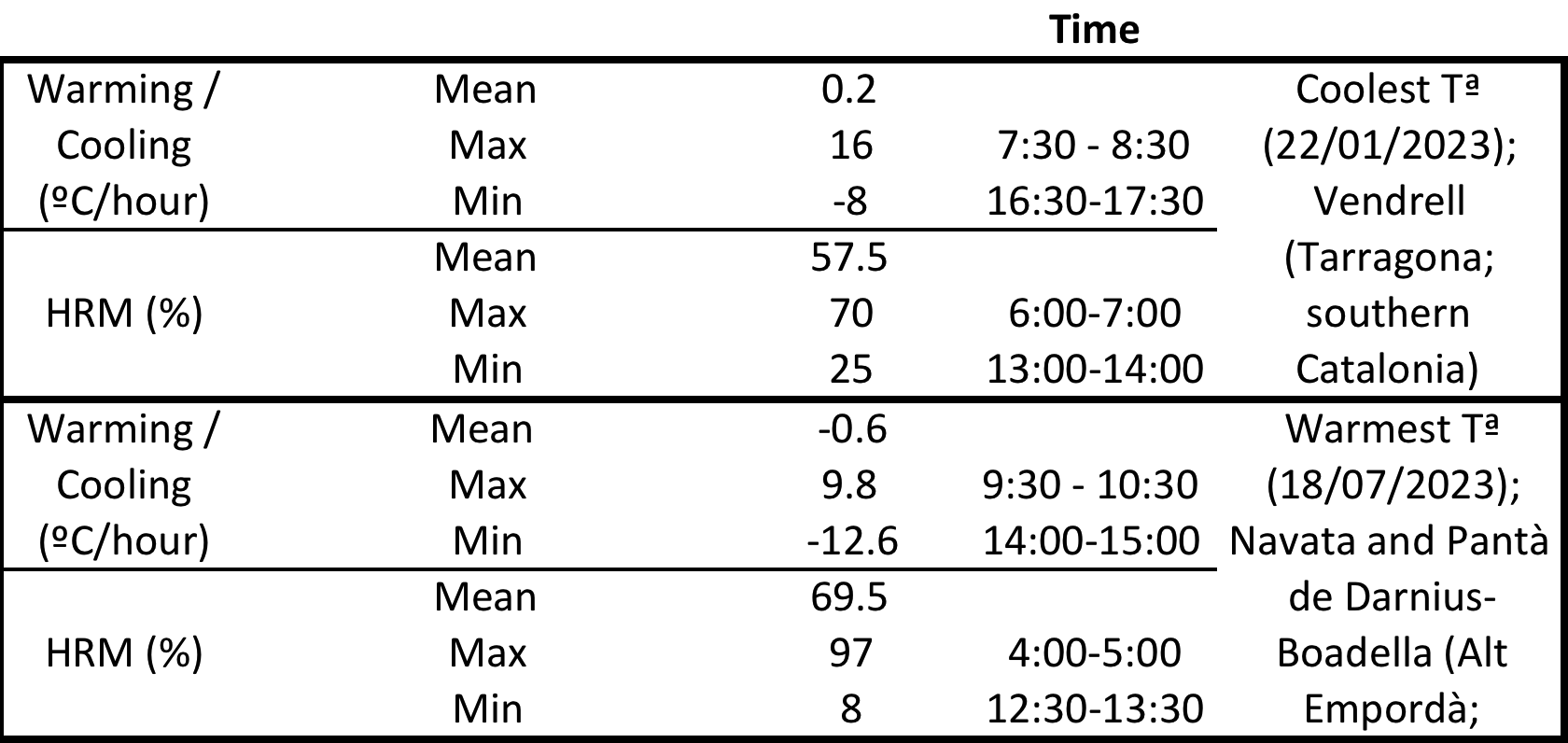

Supplement: Supplementary file 1 — (DOCX 1.06 MB) [file 11356_2026_37699_MOESM1_ESM.docx]
